# Mechanisms and the strategy for remission of type 2 diabetes mellitus

**DOI:** 10.1111/jdi.13948

**Published:** 2023-01-10

**Authors:** Tien‐Jyun Chang

**Affiliations:** ^1^ Department of Internal Medicine National Taiwan University Hospital Taipei Taiwan; ^2^ School of Medicine, College of Medicine National Taiwan University Taipei Taiwan

## Abstract

Type 2 diabetes is no longer seen as being an irreversible natural course, accompanied by progressive beta cell failure and various chronic diabetes related complications. In contrast, remission can be achieved through a personalized approach. It is a paradigm shift in our understanding of type 2 diabetes and it may be necessary to change the concept of type 2 diabetes as an urgent condition requiring rapid intervention rather than a chronic progressive disease.

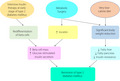

Observational studies have shown that a progressive decline of beta cell function occurs in most patients with type 2 diabetes mellitus who need increasing amounts of antidiabetic drugs. By about 10 years after diagnosis, about 50% of patients require insulin therapy to achieve appropriate blood glucose control. However, the normalization of blood glucose levels can be achieved and sustained without antidiabetic agents in some patients, and the phenomenon has become more frequent with recently updated treatments. Several studies have shown that patients with type 2 diabetes mellitus receiving metabolic surgery, intensive therapeutic interventions, or significant lifestyle modification can achieve a durable normoglycemic state^1^, ^2^, ^3^. A return to nearly normal glycemic regulation after these interventions is most likely early in the course of type 2 diabetes mellitus, and a partial recovery of both insulin secretion and insulin action was also observed. In 2009, a consensus statement initiated by American Diabetes Association (ADA) suggested that ‘remission’ signifies abatement or disappearance of the signs and symptoms of hyperglycemia^4^. Three categories of remission were proposed. ‘Partial remission’ was defined as hyperglycemia below diagnostic thresholds for diabetes [HbA_1c_ < 6.5% (48 mmol/mol) and/or the fasting plasma glucose (FPG) 100–125 mg/dL (5.6–6.9 mmoL/L)] had been maintained without antidiabetic agents for 1 year. ‘Complete remission’ was defined as normal glucose levels [HbA_1c_ < 5.7% (39 mmol/mol) and FPG < 100 mg/dL (5.6 mmol/L)] without antidiabetic agents maintained for 1 year. ‘Prolonged remission’ was defined as complete remission persisting for 5 years or more without antidiabetic agents. The update consensus on this issue was proposed in 2021, and ‘remission of type 2 diabetes mellitus’ was defined as a HbA_1c_ level < 6.5% (48 mmol/mol) measured at least 3 months after the cessation of antidiabetic agents^5^.

Previously, it was postulated that beta cells undergo apoptosis and progressively die in the worsening course of diabetes. However, in recent years the concept of dedifferentiation was proposed, and it will become the basis for strategies to revitalize beta cells and to restore function^6^.

In patients with type 2 diabetes mellitus receiving metabolic surgery, glycemic control improves rapidly within days, which occurs too quickly to account for the body weight loss alone. The change caused by food bypassing the proximal part of the small intestine is called the foregut hypothesis, and the change caused by the distal end of small intestine is called the hindgut hypothesis^7^. The intestinal hormones, incretins, play a major role in these changes. Glucagon‐like peptide 1 (GLP‐1) and gastric inhibitory polypeptide (GIP) are two incretins thought to have a significant effect.

Weight loss and consequent visceral fat loss are keys for the remission of type 2 diabetes mellitus. It has been reported that 16% of body weight loss was accompanied by a 30% intra‐abdominal fat reduction and a 65% intrahepatic fat triglyceride loss. In 2018, Taylor^8^ proposed the twin cycle hypothesis. It postulated that type 2 diabetes mellitus is caused mainly by excess, yet reversible, fat accumulation in the liver and pancreas. Excess hepatic fat worsens hepatic responsiveness to insulin and leads to increased glucose production with fasting hyperglycemia. The ectopic fat disposition in the pancreas leads to beta cell failure due to fat‐induced metabolic stress. The removal of excess fat from these organs through substantial weight loss can normalize hepatic insulin sensitivity, and is associated with beta cell recovery of acute insulin secretion after glucose challenge in the early years after the diagnosis of type 2 diabetes mellitus, which is possibly attributed to redifferentiation of beta cells. Taylor^8^ also proposed the concept of a personal fat threshold to explain the development of type 2 diabetes mellitus in individuals with a relatively low body weight associated with a lesser capacity for subcutaneous fat storage.

The remission of type 2 diabetes mellitus is plausible by restriction of food energy to achieve a body weight loss of around 15 kg. About 50% of patients with type 2 diabetes mellitus can stop all hypoglycemic agents and gain remission of type 2 diabetes mellitus by strict food energy restriction within the first 10 years of diagnosis. There are several strategies to achieve remission of type 2 diabetes mellitus. We will introduce these strategies briefly as follows. The first is metabolic surgery for patients with type 2 diabetes mellitus with morbid obesity. Metabolic surgery includes various forms, such as an adjustable gastric band (AGB), vertical sleeve gastrectomy (SG), Roux‐en‐Y gastric bypass (RYGB), and bilopancreatic diversion (BPD). In addition to weight loss, surgical intervention is accompanied by an increment of incretin hormone caused by changes in the intestinal structure to achieve favorable effects on glycemic control. The highest type 2 diabetes mellitus remission rate is BPD, then RYGB, SG, or AGB in descending order. The subjects receiving RYGB were twice as likely to achieve diabetes remission than AGB even after adjusting for body weight change^9^. According to the previous meta‐analysis, the overall type 2 diabetes mellitus remission rate after metabolic surgery is 78%^9^. Body weight is regained in some patients, and the glycemic control may worsen again. The second is intensive insulin therapy in patients with newly diagnosed type 2 diabetes mellitus with severe hyperglycemia. Initial intensive insulin therapy for 2–3 weeks in patients with newly diagnosed type 2 diabetes mellitus with uncontrolled hyperglycemia achieved remission in several studies. Type 2 diabetes mellitus remission after initial intensive insulin therapy has been reported to last for more than 2 years, and the shorter the interval between intensive insulin therapy and diagnosis, the greater the likelihood of remission^2^. Conservation of beta cell function was also confirmed after early intensive insulin therapy in patients with newly diagnosed type 2 diabetes mellitus. The third is the very‐low‐calorie diet (VLCD), which showed the most significant body weight loss. VLCD is a diet containing 800 Kcal or less per day with a relatively high protein‐to‐calorie ratio and with essential micronutrients. This diet is usually served in liquid form for 3–4 months. In the primary care‐led weight management for the remission of type 2 diabetes mellitus (DiRECT) study, VLCD combined with primary care confirmed remission in 46% of study subjects^10^. In this study group, the greater the body weight reduction, the higher the type 2 diabetes mellitus remission rate. In subjects with a greater than 15 kg body weight reduction, the remission rate achieved was 86%^10^.

In the recent decade, some new antidiabetic drugs caused significant body weight loss. Sodium‐glucose cotransporter‐2 (SGLT2) can reduce body weight and visceral fat due to urinary glucose excretion leading to caloric loss. A recent clinical trial demonstrated that a short‐term intensive intervention with insulin glargine, metformin, and dapagliflozin caused more remission than that of the conventional group (24.7% vs 16.9%), and reduced the risk of diabetes recurrence by 43%^11^. Tirzepatide is the most well recognized GLP‐1/GIP dual agonist and shows clinical promise in body weight reduction, glycemic control, and potential cardiovascular benefit. In the tirzepatide vs insulin glargine in type 2 diabetes and increased cardiovascular risk (SUPRASS‐4) clinical trial^12^, tirzepatide compared the efficacy of HbA_1c_ reduction and cardiovascular safety in type 2 diabetes mellitus patients with high cardiovascular risk inadequately controlled on oral glucose‐lowering medications. The profile of the HbA_1c_ level over time with tirzepatide treatment indicated a sustained reduction up to 104 weeks. A HbA_1c_ level of ≤ 6.5% was achieved in 66–81% of tirzepatide‐treated subjects compared with 32% with insulin glargine; and a HbA_1c_ level of < 5.7% was observed in 23–43% of terzepatide‐treated participants compared with 3% with insulin glargine. Although these new glucose‐lowering medications conferred promising perspectives in remission of type 2 diabetes mellitus, robust evidence for diabetes remission is still lacking. Further studies are warranted on the effects of diabetes remission.

The current definition of type 2 diabetes mellitus remission was based largely on expert opinion^5^, so the new criteria or different glucose standards may need to be considered. The consensus also recommends that patients achieving diabetes remission should remain under surveillance for glycemic status and diabetes complications at 1 year intervals^5^. The long‐term medical outcomes of patients achieving remission is not clear, so this issue needs further investigation. The expected duration of remission by various interventions is still not well defined, and the factors associated with the relapse of diabetes should be also studied in more detail.

In conclusion, the remission of type 2 diabetes mellitus can be achieved by metabolic surgery, intensive therapeutic interventions, or significant lifestyle modification at an early stage of type 2 diabetes mellitus with improvement of beta cell function, and the remission may be sustained for a considerable period (Figure 1). Type 2 diabetes mellitus is no longer seen as an irreversible natural course, accompanied by progressive beta cell failure and various chronic diabetes related complications. In contrast, remission can be achieved through a personalized approach. It is a paradigm shift in our understanding of type 2 diabetes mellitus and it may be necessary to change the concept of type 2 diabetes mellitus as an urgent condition requiring rapid intervention rather than a chronic progressive disease.

**Figure 1:**
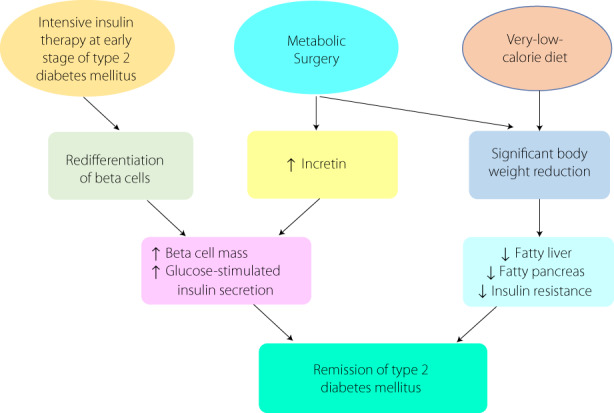
The strategies and mechanisms of type 2 diabetes mellitus remission. Intensive insulin therapy at an early stage of type 2 diabetes mellitus contributes to redifferentiation of beta cells and leads to an increased beta cell mass and potentiated glucose‐stimulated insulin secretion. Metabolic surgery leads to an elevation of plasma incretin level and significant body weight reduction, which leads to an increase of beta cell mass and potentiates glucose‐stimulated insulin secretion, ameliorates fatty liver, fatty pancreas, and insulin resistance. All of the above mechanisms contribute to remission of type 2 diabetes mellitus.

## DISCLOSURE

The author declares no conflict of interest.
